# Improving the continuity and coordination of ambulatory care through feedback and facilitated dialogue—a study protocol for a cluster-randomised trial to evaluate the ACD study (Accountable Care in Germany)

**DOI:** 10.1186/s13063-021-05584-z

**Published:** 2021-09-15

**Authors:** Leonie Sundmacher, Ronja Flemming, Verena Leve, Isabel Geiger, Sebastian Franke, Thomas Czihal, Clemens Krause, Birgitt Wiese, Frank Meyer, Matthias Brittner, Johannes Pollmanns, Johannes Martin, Paul Brandenburg, Annemarie Schultz, Emmanuelle Brua, Udo Schneider, Olga Dortmann, Christoph Rupprecht, Stefan Wilm, Wiebke Schüttig

**Affiliations:** 1 Department of Health Economics, Technical University of Munich, Georg-Brauchle-Ring 60/62, 80992 München, Germany; 2 Department of Health Services Management, Ludwig-Maximilians-University Munich, Geschwister-Scholl-Platz 1, 80539 München, Germany; 3 Institute of General Practice of Heinrich-Heine University in Düsseldorf, Moorenstr. 5, 40225 Düsseldorf, Germany; 4 Central Institute for SHI Physician Care in Germany (Zi), Salzufer 8, 10587 Berlin, Germany; 5 Institute of General Practice, Hannover Medical School, Carl-Neuberg-Str.1, 30625 Hannover, Germany; 6 Regional Association of Statutory Health Insurance Physicians Westphalia Lip, Robert-Schimrigk-Str. 4-6, 44141 Dortmund, Germany; 7 Regional Association of Statutory Health Insurance Physicians North Rhine, Tersteegenstraße 9, 40474 Düsseldorf, Germany; 8 Regional Association of Statutory Health Insurance Physicians Schleswig Holstein, Bismarckallee 1-6, 23795 Bad Segeberg, Germany; 9 Regional Association of Statutory Health Insurance Physicians Hamburg, Humboldtstraße 56, 22083 Hamburg, Germany; 10 Techniker Krankenkasse, Bramfelder Straße 140, 22305 Hamburg, Germany; 11 AOK Health Insurance Rhineland/Hamburg, Kasernenstr. 61, 40213 Düsseldorf, Germany

**Keywords:** Health care, Networks, Ambulatory care, Feedback, Cluster-randomised trial, Quality circles, Quality of care, Collaboration, Coordination, Continuity

## Abstract

**Background:**

Patients in Germany are free to seek care from any office-based physician and can always ask for multiple opinions on a diagnosis or treatment. The high density of physicians and the freedom to choose among them without referrals have led to a need for better coordination between the multiple health professionals treating any given patient. The objectives of this study are to (1) identify informal networks of physicians who treat the same patient population, (2) provide these physicians with feedback on their network and patients, using routine data and (3) give the physicians the opportunity to meet one another in facilitated network meetings.

**Methods:**

The Accountable Care Deutschland (ACD) study is a prospective, non-blinded, cluster-randomised trial comprising a process and economic evaluation of informal networks among 12,525 GPs and office-based specialists and their 1.9 million patients. The units of allocation are the informal networks, which will be randomised either to the intervention (feedback and facilitated meetings) or control group (usual care). The informal networks will be generated by identifying connections between office-based physicians using complete datasets from the Regional Associations of Statutory Health Insurance (SHI) Physicians in Hamburg, Schleswig Holstein, North Rhine and Westphalia Lip, as well as data from three large statutory health insurers in Germany. The physicians will (a) receive feedback on selected indicators of their own treatment activity and that of the colleagues in their network and (b) will be invited to voluntary, facilitated network meetings by their Regional Association of SHI physicians. The primary outcome will be ambulatory-care-sensitive hospitalisations at baseline, at the end of the 2-year intervention period, and at six months and at 12 months after the end of the intervention period. Data will be analysed using the intention-to-treat principle. A pilot study preceded the ACD study.

**Discussion:**

Cochrane reviews show that feedback can improve everyday medical practice by shedding light on previously unknown relationships. Providing physicians with information on how they are connected with their colleagues and what the outcomes are of care delivered within their informal networks can help them make these improvements, as well as strengthen their awareness of possible discontinuities in the care they provide.

**Trial registration:**

German Clinical Trials Register DRKS00020884. Registered on 25 March 2020—retrospectively registered.

**Supplementary Information:**

The online version contains supplementary material available at 10.1186/s13063-021-05584-z.

## Administrative information

**Table:** 

Title	Improving the continuity and coordination of ambulatory care through feedback and facilitated dialogue – a study protocol for a cluster-randomised trial to evaluate the ACD study (Accountable Care in Germany)
Trial registration	Trial registration DRKS00020884 German Clinical Trials Register (also WHO trial registration with the same registration number)
Protocol version	Issue Date: 11^th^ November 2020 Version 1
Funding	The study is fully funded by the German Federal Joint Committee - GBA (Grant no. 01VSF16046 (ACD)).
Author details	^1^ Department of Health Economics, Technical University of Munich ^2^ Department of Health Services Management, Ludwig-Maximilians-University Munich ^3^ Institute of General Practice of Heinrich-Heine University in Duesseldorf ^4^ Central Institute for SHI Physician Care in Germany ^5^ Institute of General Practice, Hannover Medical School ^6^ Regional Association of Statutory Health Insurance Physicians Westphalia Lip ^7^ Regional Association of Statutory Health Insurance Physicians North Rhine ^8^ Regional Association of Statutory Health Insurance Physicians Schleswig Holstein ^9^ Regional Association of Statutory Health Insurance Physicians Hamburg ^10^ Techniker Krankenkasse (Statutory Health Insurer) ^11^ AOK Health Insurance Rhineland/ Hamburg (Statutory Health Insurer)
Name and contact information for the trial sponsor	Leonie Sundmacher, Ph.D., Department of Health Economics, Technical University of Munich Wiebke Schüttig, Ph.D., Department of Health Services Management, Ludwig-Maximilians-University Munich email: leonie.sundmacher@tum.de
Role of sponsor	The funding source has no influence over the study design, implementation, evaluation or dissemination of the findings of the study.

## Background: study objectives and trial design

Office-based physicians and psychotherapists in Germany see about 70 million patients each year for a total of 553 million cases [1]. More than 85% of all patients are treated by more than one physician. Overall, 54,819 GPs and 94,891 specialists were accredited in 2019 to treat patients enrolled in statutory health insurance (SHI) [2], which covers almost 90% of the population in Germany. Almost all GPs and about half of all specialists in Germany work in office-based practices.

Patients in Germany are free to seek care from any of these physicians. There is no nationwide system of gatekeeping that regulates their access to office-based specialists, and they can always seek multiple opinions on a diagnosis or treatment. The high density of physicians and the freedom to choose among them have led to a need for better coordination between the multiple health professionals treating any given patient. This has been hampered by the delayed introduction of an electronic health card for patients and a consequent lack of structured documentation of the care they have received.

Ensuring continuity of care is therefore one of the greatest challenges in the German health system, particularly for patients with chronic diseases. Studies suggest that a lack of knowledge about a patient’s treatment history can lead to polymedication, duplicate testing and inadequate follow-up care after discharge from hospital [3–5]. Gaps in care after discharge and in ambulatory follow-up, such as non-adherence to pharmacological treatment prescribed in hospital, a lack of timely medication review and missed follow-up visits, increase the risk of hospital readmission [6, 7]. Moreover, there is evidence that good continuity of ambulatory care can reduce the overall risk of hospitalisation, particularly of that due to ambulatory-care-sensitive indications [8–10]. It has been assumed that such hospitalisations can be reduced by treating acute conditions and managing chronic illnesses more effectively, and by increasing the uptake of immunisation against infectious diseases [11]. At the same time, Cochrane reviews have confirmed that providing feedback on selected indicators can improve everyday medical practice by shedding light on previously unknown relationships [12, 13].

### Study objectives

We hypothesise that providing office-based physicians with comprehensive information on their patients’ treatments and diagnoses, and giving these physicians the opportunity to discuss this information with colleagues who treat the same patients, might improve care coordination and patient outcomes in the ambulatory care sector.

In Germany, physicians do not necessarily learn about the other treatments or diagnoses unless the patient tells them or asks explicitly for their records to be transferred from one doctor to another. Analysing large, merged data sets from Regional Associations of SHI Physicians (ASHIP) and large statutory health insurers allows us to follow patient pathways, including all appointments, treatments, medications and diagnoses across the health system over time. The objectives of our study are to use this information to (1) identify informal networks of physicians who treat the same patient population, (2) provide the physicians with feedback on their network and patients based on routine data and, (3) give the physicians the opportunity to meet one another in facilitated network meetings.

We will use the routine data described above to identify informal networks of physicians who provided care to the same patients with chronic diseases during a specified period. The physicians in these networks will receive feedback on selected indicators of per-physician treatment activity in their network and the care received by patients. Subsequently, the physicians will be invited to voluntary, facilitated network meetings during which they will be able to use the information provided to them to discuss the challenges of coordinating care in everyday medical practice and can jointly agree upon clinical pathways for selected indications. These networks will be compared to informal networks that did not receive additional information or invitations to meetings and that provide usual care.

### Study design

The ACD study is a non-blinded, cluster-randomised study (with unequal cluster size) of informal networks of GPs and office-based specialists and their patients. The units of allocation are the informal networks, which will be randomised to the intervention (i.e., feedback and facilitated meetings) and control group (usual care) using 1:1 randomisation.

## Methods: study setting, participants, randomisation, intervention and outcomes

### Study setting, network generation and participants

The informal networks will be generated by identifying connections between office-based physicians using complete datasets from the Regional Associations of SHI Physicians in Hamburg, Schleswig Holstein, North Rhine and Westphalia Lip. The datasets are collected for administrative or billing purposes.

A connection between two physicians will be defined as the sharing of at least 20 patients who, for at least one of the two physicians, account for 5% or more of the total number of patients he or she sees within the observation period. Communities within networked structures will be generated using a modularity optimisation algorithm. A modularity signals that there are more connections than expected in a community. The networks will include a minimum of 20 and a maximum of 120 physicians.

Office-based specialists who are not frequently involved in the care of patients with chronic disease or who do not have direct patient contact will be excluded from the analysis. These physician groups are paediatricians (see also the section on ‘participants’), laboratory physicians, microbiologists, virologists and infection epidemiologists, maxillofacial surgeons, pathologists, radiologists, neuroradiologists, radiation therapists, transfusion physicians and child and adolescent psychotherapists.

**Fig. 1:**
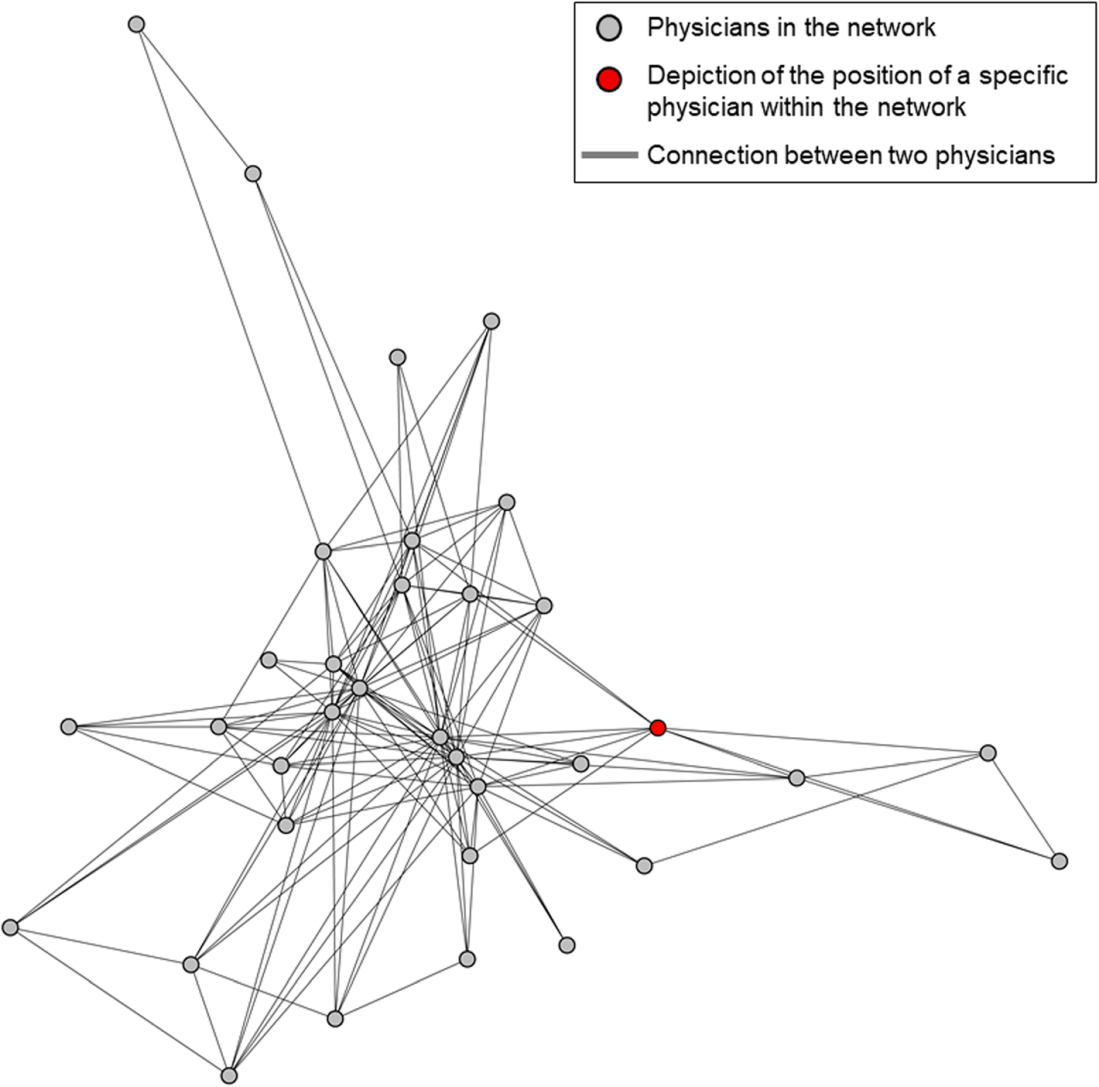
A network identified among office-based physicians

Figure 1 shows an example of a network identified among office-based physicians.

The analysis sample that will be used to identify the informal networks of office-based physicians will include only those patient contacts for which we assume health outcomes can be improved through (1) continuity of care in the ambulatory sector, (2) effective coordination between office-based physicians, (3) effective acute treatment in the case of vulnerable patients or (4) any combination of these. In a team with physicians from Düsseldorf Medical School, the Regional Association of SHI Physicians Hamburg and the AOK Rhineland/Hamburg, we will identify patients with chronic diseases who are often hospitalised due to ambulatory-care-sensitive conditions [14]. These are patients with ischaemic heart diseases (ICD I20–I25), heart failure (I50), other diseases of the heart and circulatory system (I05, I06, I09, I08, I49, I48, I67, I70, I73, I78, I80, I83, I87, I95, R00, I42, I74), chronic obstructive pulmonary disease (J44, J47), mental and behavioural disorders due to use of alcohol and opioids (F10, F11), dorsopathies (M42, M47, M53, M54, M50, M51), bronchitis (J20, J21, J22, J40, J41, J42, J43), hypertension (I10–I15), gastroenteritis and certain diseases of the intestine (K52, K57, K58, K59), intestinal infectious diseases (A00–A09), influenza and pneumonia (J10–J16, J18), infections of the ear, nose and throat (H66, J01–J04, J06, J31, J32, J35, H65, H73, R07.0), depressive disorders (F32, F33), diabetes mellitus (E10, E11, E13, E14, E16) or gonarthrosis (M17). We will assume that a patient had one of these diseases if the patient record contains the same diagnosis in at least two different quarters in a year. Exceptions are diagnoses of bronchitis, gastroenteritis, intestinal infectious diseases, influenza and pneumonia, and infections of the ear, nose and throat. For these diseases, a confirmed diagnosis in any single quarter of a year was sufficient. Dialysis patients and patients aged 17 years and younger will be excluded from the analysis. The networks will be generated by the Department of Health Services Management at LMU Munich and the Central Institute for SHI Physician Care in Germany.

### Intervention

**Fig. 2:**
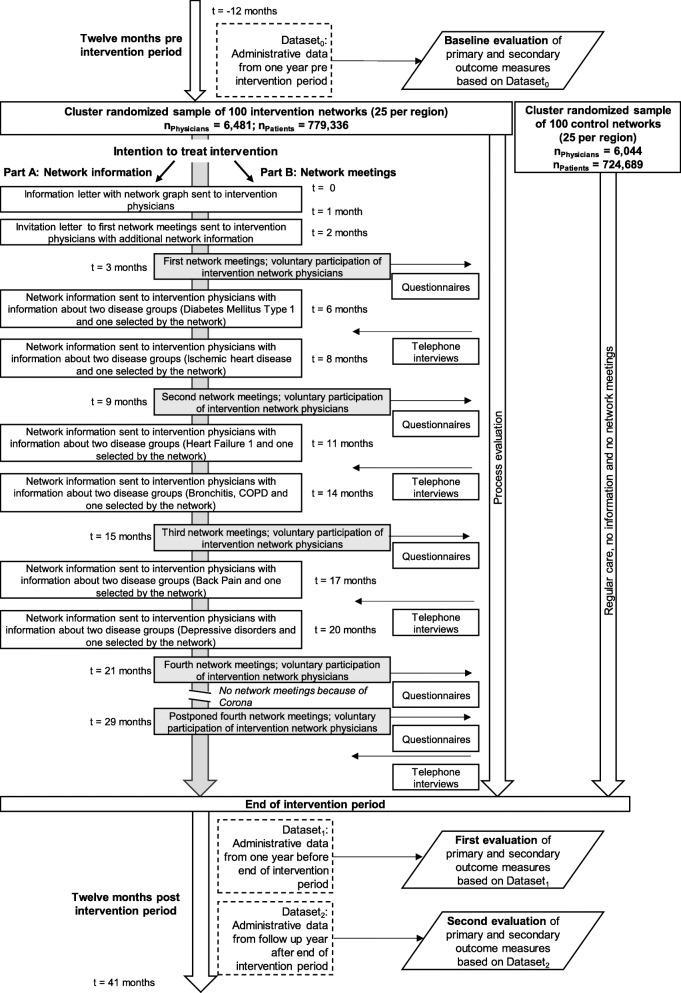
Intervention and methods of the ACD study

The physicians in each intervention network will receive feedback sheets and be invited by their respective Regional Associations of SHI Physicians to take part in a series of four voluntary network meetings. The meetings will take place in months 3, 9, 15 and 21 of the intervention. The invitations will be sent out before each meeting and followed by one reminder in cases where no reply is received. In total, the physicians will be sent eight feedback sheets. These will be sent out at least 2 weeks before each meeting. The meetings will be led by a trained facilitator. The 2-year intervention will take place from late 2018 to late 2020. (Addendum October 2020: Due to the COVID-19 pandemic beginning in early 2020, we had to cancel all network meetings between March and May 2020. The meetings were resumed in June 2020 and will be held until January 2021). Figure 2 summarises the study design, intervention and methods of the ACD study.

The feedback sheets will contain (a) information on the characteristics of the networks and (b) indicators of the quality of patient care for the diseases considered in the analysis. The former, (a), will include the following: a graphical representation of each network showing the physician’s own position within it (see red vertex in Fig. 1); the number and composition of the physicians and physician groups; an indication of the strength of the connections between the physicians; and information on the frequency of diagnoses and multimorbidity among patients receiving care from the network in comparison to statistics of neighbouring networks and regional averages. In the latter, (b), the indicators of the quality of patient care will relate to the disease groups described above and be selected based on the results of a comprehensive literature search, the availability of routine data, and an evaluation by the medical members of the study team. Overall, we estimate that over 200 indicators for disease groups will be calculated and that most of these will be process indicators. For the sake of brevity and to facilitate discussion, each feedback sheet will contain information only on two disease groups and show the process indicators always in relation to the statistics of neighbouring networks and regional averages.

During each meeting, the physicians will be given the opportunity to discuss the results of the feedback sheets, with the discussion facilitated in such a manner that they will focus on how best to achieve continuity of care. Additionally, they will be given the opportunity to develop a patient pathway for one of two diseases discussed during each meeting. At the end of each meeting, the physicians will decide which two disease groups they would like to see on the next feedback sheet and which diseases they would like to discuss in the upcoming network meeting. In one of the network meetings, the physicians will also receive detailed information on typical treatment pathways that patients within a disease group have followed within the network.

The indicators of the quality of patient care will be calculated based on data from the Regional SHI Associations, the AOK Health Insurance Rhineland/Hamburg, the AOK Health Insurance North West and the Techniker Krankenkasse. Together, these statutory health insurers cover 41.49% of the population in the regions in which the intervention will take place.

Before the series of four network meetings begins, the facilitators will take part in training sessions organised by the Institute of General Practice at Heinrich-Heine-Universität in Düsseldorf. They will be able to partake in additional training during the study period. Each facilitator will receive a manual that describes the aims and steps of the intervention.

### Outcomes

The primary outcome for measuring potential improvements in continuity and coordination of care in the intervention networks compared to the control networks will be hospital cases resulting from ambulatory-care-sensitive diagnoses. The assumption underlying ambulatory-care-sensitive diagnoses (ACSD) is that effective treatment of acute conditions, good management of chronic illnesses and immunisation against infectious disease can reduce the risk of specific types of hospitalisations [11]. The analysis sample will include only those patients who have been diagnosed with a condition that can potentially lead to one of the 14 most common hospitalisations for ACSD in Germany (please also see the section on ‘participants’). Table 1 shows the most common diagnoses for ambulatory-care-sensitive hospitalisations (ACSH) in Germany taken from a recent review and update of ACSH [14].

**Table 1:** List of ambulatory-care sensitive-hospitalisations (including ICD-10 disease codes) for the primary outcome measure

No.	Disease group	ICD-10
1	Ischaemic heart diseases	I20, I25.0, I25.1, I25.5, I25.6, I25.8, I25.9
2	Heart failure	I50
3	Other diseases of the circulatory system	I05, I06, I08.0, I49.8, I49.9, I67.2, I67.4, I70, I73, I78, I80.0, I80.80, I83, I86, I87, I95, R00.0, R00.2, R47.0
4	Bronchitis and COPD	J20, J21, J40-J44, J47
5	Mental and behavioural disorders due to use of alcohol or opioids	F10, F11
6	Back pain [dorsopathies]	M42, M47, M53, M54
7	Hypertension	I10-I15
8	Gastroenteritis and other diseases of intestines	K52.2, K52.8, K52.9, K57, K58, K59.0
9	Intestinal infectious diseases	A01, A02, A04, A05, A07-A09
10	Influenza and pneumonia	J10, J11, J13, J14, J15.3, J15.4, J15.7, J15.8, J15.9, J16.8, J18.0, J18.1, J18.8, J18.9
11	Ear nose throat infections	H66, J01-J03, J06, J31, J32, J35
12	Depressive disorders	F32, F33
13	Diabetes mellitus	E10.2-E10.6, E10.8, E10.9, E11, E13.6, E13.7, E13.9, E14, E16.2
14	Gonarthrosis [arthrosis of knee]	M17.0, M17.1, M17.4, M17.5, M17.9

To ensure the reliability of the primary outcome, a patient will be reported as having a relevant ACSH if this was the main diagnosis of his or her hospital stay. Secondary diagnoses will not be considered.

The secondary outcome will be rates of treatment provided in accordance with the relevant clinical practice guidelines. Treatment in this context will be defined as (a) the type and amount of medication prescribed by physicians in the networks, (b) whether regular follow-up visits have taken place and (c) whether the physicians follow the recommended diagnostic procedure [15–19].

Both the primary and secondary outcomes will be calculated based on the routine data described above and evaluated at baseline, at the end of the 2-year intervention period, and at 6 months and at 12 months after the end of the intervention period. The outcomes will be evaluated using an intention-to-treat approach within multi-level models [20].

Additionally, in accordance with MRC guidelines [21], we will undertake a process evaluation and a cost-effectiveness analysis. Through the latter, it will be possible to evaluate how changes in the relevant medical effects of the intervention are related to changes in its costs [22]. The analysis will take account both of additional costs from the perspective of the statutory health insurers (payer perspective) and the marginal effects of hospitalisation rates among the intervention networks compared to those among the control networks. This information will be used to discuss the potential to implement the intervention to other contexts within Germany [21].

The process evaluation will combine qualitative and quantitative approaches to identify potential causal factors, as well as enablers of and barriers to the acceptance of feedback and the implementation of facilitated network meetings throughout the study. Initially, all 60 facilitators will have to complete a questionnaire on their expectations of and satisfaction with the ACD-specific training they receive. After each network meeting, participants and facilitators will be asked to fill in a questionnaire to reflect on the meeting and their personal experiences of the ACD study. Additionally, 36 semi-structured telephone interviews will be held with facilitators and randomly selected physicians who give consent to be contacted. After receiving at least two network information sheets, participating physicians will be offered the chance to give feedback on the layout and content of the network information. Finally, coordinators from the Regional Associations of SHI Physicians, who were responsible for organising the network meetings, were asked for written feedback on important organisational aspects of the intervention and for lessons learnt during the study.

(Addendum October 2020: Due to the COVID-19 pandemic and the resulting cancellation of meetings between March and May of 2020, all physicians from the intervention group received a questionnaire for them to assess the intervention, including questions on their satisfaction with the feedback sheets and organisation of network meetings and to make suggestions for improvement.)

### Randomisation and sample size estimation

Among the networks identified in the data set described above, we will select a random sample of 100 intervention and 100 control networks. These networks will be divided into strata according to (a) the four regions covered by the Regional Associations of SHI Physicians and (b) whether they are located in an urban or rural area, as it can be assumed that the structure of the networks will differ depending on their location, which, in turn, will have an impact on the measured outcomes [23]. Within these strata, the networks will be randomised to the intervention and control groups with equal size [24]. Networks of the strata will be randomly assigned to the intervention or control group by statisticians of the Hannover Medical School using the software nQuery 7.0. The statisticians are not otherwise involved in the planning of the study design. Sequence allocation and assignment is conducted based on the network identifier (generated by the study group) without information on physician identities.

The sample size estimation based on the assumption of a 20% hospitalisation rate and a 15% reduction in ACSHs following the intervention, we thus expect a 17% ACSH rate in the intervention group. The numbers were chosen after reviewing comparable studies and analysing hospital routine data [14, 25, 26]. With a 5% significance level and a power of 80%, 2629 patients per group would be needed to show this effect (2-sided *χ*^2^ test). For cluster-randomised studies, the estimated sample size must be adjusted by the design effect, which is a function of cluster size and the intracluster correlation coefficient (ICC). Assuming an ICC of 0.015 [27] and an average of 300 patients per network yields a design effect of 5.485. This means that the estimated number of cases of an individual-randomised study has to be multiplied by 5.485. With this design effect, 28,840 patients and thus 96 networks per arm (intervention and control) will be needed.

As described above, the intervention networks will receive feedback sheets and be invited to facilitated network meetings by the Regional Association of SHI physicians. According to Section 287 of Book V of the German Code of Social Law (§ 287 SGB V) the regulatory authorities confirmed that Regional Associations of SHI Physicians may send the feedback sheets to their members without prior written, informed consent by the office-based physicians (please see section on ‘participant consent’). Participation in the informal network meetings will, however, be voluntary. Data will be analysed using an intention-to-treat approach, with all office-based physicians who receive feedback sheets being considered in the evaluation, regardless of whether they participate in the voluntary network meetings. This approach will help avoid biased estimates due to the self-selection of physicians who are more enthusiastic about taking part in the network meetings [28]. Furthermore, we will slightly oversample the informal networks (100 instead 96) in case all physicians in an informal network formally reject the feedback sheets sent by the Regional Association of SHI physicians.

## Data management, statistical methods and ethics

### Data management and approval by regulatory authorities

Our application to use administrative data for the ACD study was granted by the regulatory authorities of the Regional Association of SHI Physicians in North Rhine (State Ministry of Employment, Health and Social Affairs in North Rhine Westphalia, granted 2017/11/21), Westphalia Lip (State Ministry of Employment, Health and Social Affairs in North Rhine Westphalia, granted 2017/11/21), the Hamburg Senate for Health and Consumer Protection (granted 2018/02/15) and Schleswig Holstein (State Ministry of Health, Youth, Families and Seniors in Schleswig Holstein, granted 2017/09/29)), as well as by AOK Rhineland/Hamburg and AOK North West (Ministry of Employment, Health and Social Affairs in North Rhine Westphalia, granted 2017/10/24), and the Techniker Krankenkasse (Federal Office for Social Security, granted 2017/09/22). The state ministries are the responsible regulatory authorities in the states. The Techniker Krankenkasse, which provides statutory insurance on a nationwide basis, is supervised by the Federal Office for Social Security.

**Fig. 3:**
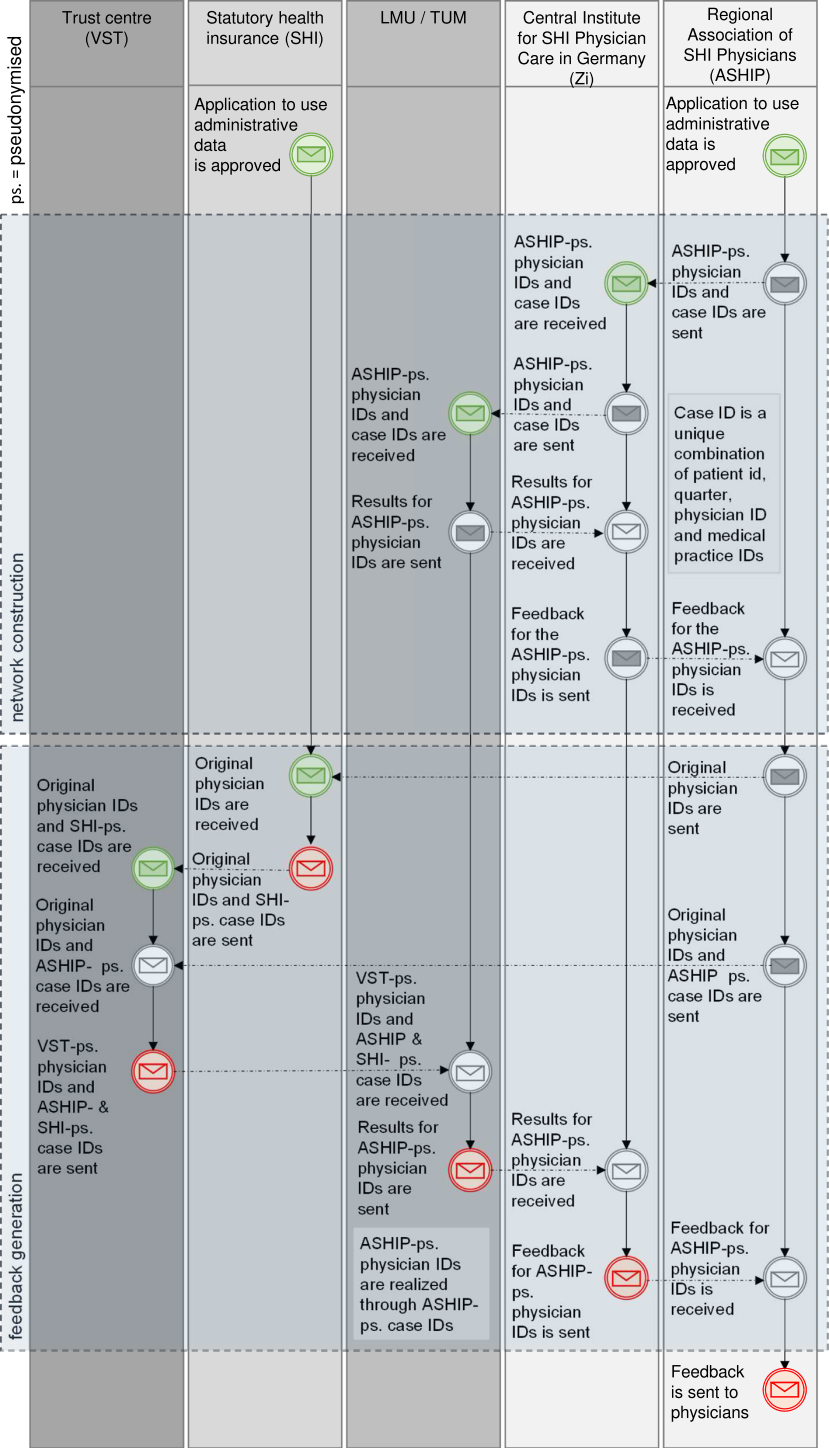
Data flow and pseudonymisation in the ACD study

Following our application to the relevant regional regulatory authorities in accordance with Section 75 of Book X of the German Code of Social Law (§75 SGB X), data from the Regional Associations of SHI Physicians and the statutory health insurers will be linked within a Trust Centre. The data linkage and the pseudonymisation are shown in Fig. 3.

The Regional Associations of SHI Physicians will use the same tool to pseudonymise the patient IDs, physician IDs and medical practice IDs. The tool uses a hash-algorithm to generate 40-digit pseudonyms. All IDs are extended by a “secret” prior to the hashing. This secures the pseudonyms against random hits when attempting re-pseudonymisation using brute force methods. The pseudonymised data are then sent via an SFTP server provided by the Central Institute for SHI Physician Care in Germany (Zi), who will combine the physician association data by regions before forwarding them to the analysts. Furthermore, to enable the data linkage, the Regional Associations of SHI Physicians will send lists (key lists) of un-pseudonymised physician IDs to the Trust Centre. With these lists, the Trust Centre can check whether physicians have been pseudonymised correctly.

The statutory health insurers will send their data via a secure data server connection to the Trust Centre. The patient data will be pseudonymised, and the physician’s office and hospital IDs will be sent un-pseudonymised. The Trust Centre uses a reversible dual technique to pseudonymise the physician data. The IDs of physicians who are part of the Regional Associations of SHI Physicians will be pseudonymised/replaced with the pseudonym from the key lists. Lastly, the pseudonymised data will be secured in a VeraCrypt container and transferred via external hard drives to the analysts. Analysts will have information on the allocation of physician pseudonyms to intervention and control networks. For the analysis, the data will be placed on a separate server and kept in a secure VeraCrypt container, accessible to the analysts only.

The data provided by the Regional Associations of SHI Physicians encompass all patient contacts in the ambulatory care sector that are billed to the statutory health insurer except for some cross-sectoral services and services purchased through selective contracting. The data provided by the statutory health insurers encompass all patient contacts billed to participating statutory health insurers, including contacts in hospitals. The data are collected for billing purposes and are therefore routinely subject to thorough quality control by the data holder. Additional quality checks of the data will be conducted by the Trust Centre, the Central Institute for SHI Physician Care in Germany and the analysts. These quality checks will include summary statistics of variables and the systematic comparison of different data tables.

An external data monitoring committee was not established in the study due to the non-invasive character of the study with no critical safety concerns for patients or physicians. Instead, a project board was established. This board consisted of one representative of the project management, one for the scientific project partners, one for the associations of statutory health insurance physicians, and one for the health insurance funds. The board holds biannual conferences on the status of the project.

### Statistical methods

The networks will be identified in an iterative fashion. Patients shared by the office-based physicians will be arranged into a matrix that will be used to generate the network structure. This will subsequently be divided into intervention networks using the modularity optimising multilevel algorithm of the igraph package in R. The indicators for the feedback sheet will comprise the characteristics of the networks and the underlying patient population, which will be summarised using descriptive statistics. The indicators of quality of patient care will be calculated as combined indicators encompassing medication, procedures, use of services and/or (re-)admissions.

For the evaluation, we will analyse differences in the primary and secondary outcomes between the intervention and control groups. Despite randomisation, it is conceivable that the results might be affected by external factors, such as the individual disease risk of patients, itself driven by differences, for example, in demographics [29], compliance [30], socioeconomic factors [31], and the organisation of the hospital sector [32]; this will therefore be adjusted using multilevel regression models with the network as random effect.

Comprehensive sensitivity analyses of the evaluation outcomes are planned, including systematic comparisons of competing models of risk adjustment [33] and different levels of significance.

For the cost-effectiveness analyses, the cost data will be taken from the routine data provided by the three participating statutory health insurers (Techniker Krankenkasse, AOK Rhineland/Hamburg, AOK North West). We will use the German standard cost model and choose the perspective of the statutory health insurers (i.e., the payer perspective) in order to provide information for reimbursement decisions. The primary outcomes of the cost-effectiveness analyses will also be hospital admissions resulting from ambulatory-care-sensitive conditions. The incremental cost-effectiveness ratios (ICER) will be calculated using bootstrapping methods. The statistical uncertainty will be estimated using cost-effectiveness acceptability curves (CEAC).

## Discussion

Although many researchers have focused on identifying informal networks [34–36], this will be the first randomised, controlled study to (a) provide physicians within such networks with structured feedback on network characteristics and patient care based on an analysis of routine data and (b) invite these physicians to facilitated, face-to-face meetings in which they can exchange information and views on the coordination of care in the German ambulatory sector. The approach that will be taken in our intervention builds on the results of studies suggesting that health outcomes can be improved through regular, informal communication between physicians and agreed treatment pathways, including standards for timely transfer of patient-relevant data [26].

Linking routine data from the Associations of SHI Physicians and the statutory health insurers makes it possible to obtain a bird’s eye view of actual patient care pathways and the care delivered to patients, as well as to identify the physicians who deliver this care and to provide these physicians with information on treatment activity.

There is strong evidence from Cochrane reviews that feedback can improve everyday medical practice by shedding light on previously unknown relationships [12, 13]. Providing physicians with information on how they are connected with their colleagues and what the outcomes are of care delivered within their informal networks can help them make these improvements, as well as strengthen their awareness of possible discontinuities in the care they provide.

A potential limitation of this study is the willingness of office-based physicians to take part in the network meetings. Their voluntary participation is planned to take place in the evenings after their practices have closed. It is uncertain, however, whether the invitations and feedback sheets will suffice as motivation for them to take part in the meetings. In order to address this risk, the Regional Associations of SHI Physicians will publish early information about the intervention in their member newspapers and provide the names of people in the various organisations who can be contacted if their members have further questions about the study. In the process evaluation, we will analyse potential barriers to participation in the network meetings.

## Ethics and dissemination

The protocol was reviewed and approved by the ethics committee of Düsseldorf Medical School (2018/01/22 and 2017/10/24) and by the State Ministry of Employment, Health and Social Affairs in North Rhine Westphalia (2017/11/21), the State Ministry of Social Affairs (2017/09/22), Health, Youth, Families and Seniors in Schleswig Holstein (2017/09/29), the Hamburg Senate for Health and Consumer Protection (2018/02/15) and the Federal Office for Social Security (2017/09/22) in their role as regulatory authorities. Dissemination strategies will include reports, the presentation of results in publications and conferences, and public relations.

## Trial Status

The protocol has the version number 1, dated 11th November 2020, the recruitment began in July 2018 and will be completed by January 2021.

## Supplementary Information

**Additional file 1: Table S1.** SPIRIT Checklist.

## Data Availability

Dissemination strategies include reports, the presentation of results in peer-reviewed journals and at conferences, and public relations activities in line with the recommendations of the International Committee of Medical Journal Editors (ICMJE). Due to data protection restrictions and regulations only the LMU/TUM will have access to the full dataset.

## Abbreviations

ACSD: Ambulatory-care-sensitive diagnoses
ACSH: Ambulatory-care-sensitive hospitalisation
ASHIP: Regional Associations of SHI Physicians
CEAR: Cost-effectiveness acceptability curves
GKV: Statutory Health Insurance
ICC: Intracluster correlation coefficient
ICER: Incremental cost-effectiveness ratios
LMU: Ludwig-Maximilians-University Munich
SHI: Statutory Health Insurance
VST: Trust Centre
Zi: Central Institute for SHI Physician Care in Germany
TUM: Technical University of Munich
